# Aggregation in woodlice: social interaction and density effects

**DOI:** 10.3897/zookeys.176.2258

**Published:** 2012-03-20

**Authors:** Pierre Broly, Romain Mullier, Jean-Louis Deneubourg, Cédric Devigne

**Affiliations:** 1 Université Lille Nord de France, Lille, France; 2 UCLILLE, FLST, Laboratoire Environnement & Santé, Lille, France; 3 Université libre de Bruxelles, Unité d’Ecologie Sociale, Bruxelles, Belgium

**Keywords:** Woodlouse, aggregation, social interaction, density, dynamics

## Abstract

Terrestrial isopods are known to be sensitive to humidity, brightness or temperature. Until now, aggregation was assumed to depend on these sensitivities as a result of individual preferences. In this paper, we show that the social component is also important in the isopod aggregation phenomenon. In experimental arenas with two identical shelters up to nearly 90% of woodlice aggregated under shelters. This aggregation was quick as in 10 minutes most of the animals aggregated, irrespective of their density. Nonetheless, 10–15% of the animals walked around the arena, rarely forming very small and short-lasting aggregates outside shelters. Woodlice aggregated preferably under one of the shelters in 77% of experiments. Indeed, almost 80% of the animals out of 40, 60 or 80 animals in the arena aggregated under one shelter. In arenas with 100 individuals the aggregations were proportionally smaller (70%). Our results revealed that 70 animals was a maximum number of woodlice in an aggregate. We concluded that the location of aggregates is strongly governed by individual preferences but the dynamics of aggregation and collective choice are controlled by social interaction between congeners. The tested densities of the animals in the arena did not impact the aggregation patterns.

## Introduction

Woodlice are mainly detritivorous organisms feeding on leaf litter, decayed wood, fungi, and bacteria. They are one of the most important groups of organisms driving the dynamics of soil (Hassall et al. 1987, Zimmer et al. 2002). In European woodlands the density of woodlice is very variable and can reach 800 individuals/m²; however, in some calcareous grasslands their density can reach 3000 individuals/m² (Gongalsky et al. 2005, Paoletti and Hassall 1999). These measures given per m² do not really reflect densities observed at the scale of micro-habitat used by woodlice. Indeed, it is frequently observed that there is a strong variation of density between different micro-habitats in similar environments with ranges from less than 10 individuals to more than 60 congeners (Davis and Sutton 1977, Gongalsky et al. 2005, Paris 1963). The observation of such variation can be explained by the individual preferences of woodlouse in heterogeneous environments with different qualities of micro-habitats. Individual preferences of woodlouse are well known and have been strongly studied in the past (Cloudsley-Thompson and Constantinou 1987, Sutton 1972, Sutton and Holdich 1984). However, recent studies have shown the importance of social interactions in woodlice aggregation (Devigne et al. 2011). Hence, aggregation patterns observed cannot be explained only with individual preferences but they result from synergy and competition between such preferences and the social interaction between individuals. This new approach in the understanding of woodlice aggregation will permit us to better study how the individuals could be distributed in an environment. In consequence, woodlice distribution will depend on the micro-habitats available but also will depend on the density of congeners, the social interactions being density dependent. This paper aims to give new insights about the speed of the aggregation dynamics and collective choice made by groups of woodlice in standardized experimental conditions. This paper specifically deals with the impact of density of congeners on the characteristics of aggregation process.

## Methods

### Rearing conditions

The rough woodlouse, *Porcellio scaber* Latreille, 1804 is a widely distributed terrestrial isopod well known to form aggregates. Individuals were collected in the gardens of Lille Catholic University (Northern France). They were reared in terraria (410x240x225mm) on a plaster layer regularly moistened (H°=75 ±10%). They were fed with litter of maple, beech and oak leaves. Room temperature (as well as the experimental set-ups) was kept at 23 ±2°C. Photoperiod was 14:10 (L:D).

### Experimental set-up

**Figure 1.**
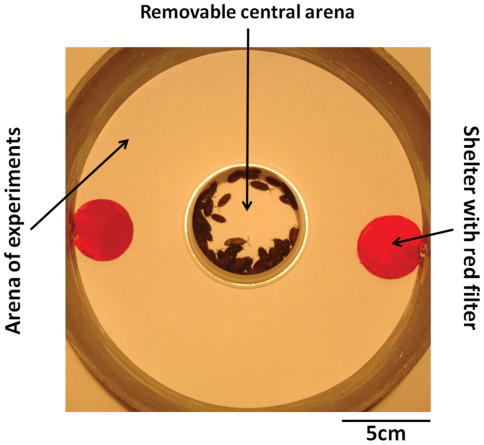
Experimental set-up.

The experimental set-up consisted of a circular arena (diameter193mm) with two dark shelters (Fig. 1). The experimental set-up was placed on a white sheet of paper which was changed between each experiment.

Shelters consisted of a small glass plate (diameter 35mm placed at 5mm of soil). Darkness in shelters was achieved by adding to glass plates, two layers of red ROSCO^®^ filters (ref. Roscolux #19 Fire – this filter changed the spectrum of light by transmitted to nearly only red energy). The set-up was lit with 156 lux and the brightness under both shelters was only 41 lux. Both shelters in the arena were strictly identical in size, darkness and contact surface with the edge of the arena. No bias between the number of woodlice observed under the left and the right shelter could be found by analyzing the whole data (Wilcoxon’s test, p=0.263, N=87).

Before the experiments, woodlice were placed in groups of 40 (N=29), 60 (N=20), 80 (N=20) or 100 (N=19) individuals in the centre of the experimental arena in a small removable central arena (diameter 65mm – Fig. 1). When the animals were calm (after about 5 minutes) the small central arena was removed and the aggregation dynamic was video-recorded during 45 minutes (thanks to a Sony camera CCD firewire - DMK 31BF03). Hence, densities used, in these experiments, ranged from 1325 to 3315 individuals/m².

### Data analysis

In order to determine whether woodlice selected one shelter preferentially, binomial tests were carried out with H_0_ assuming an equal distribution of woodlice between both shelters. After this binomial test, it is possible to define the “*winning”* shelter as being the shelter with the higher number of woodlice at the end of the experiment and the “*losing”* shelter as the other one (for the method, see Sempo et al. 2009).

X² test was used to compare the proportion of experiments with choice of one shelter according to density.

Since our data did not meet conditions for parametric tests, comparisons of results obtained with different densities were carried out with a Kruskal-Wallis test followed, if necessary, by a Dunn’s test.

GraphPad software InStat 3 was used to carry out the statistical tests.

### Results

First of all, only one of all 88 replicates did not show any aggregation during the 45 minutes of observation. Hence, this replicate was not considered. In all the other replicates, regardless of the density, nearly 90% of woodlice were observed to aggregate under shelters after 45 minutes (Table 1). No experiments showed a large aggregation outside shelters at the end of the 45 minutes. However, some woodlice (less than 15%) generally still walked around in the arena (Table 1) rarely forming very small aggregates (only two observations in the 87 experiments carried out).

**Table 1.** Proportion of aggregated woodlice and proportion of woodlice under shelters or outside shelters at the end of experiments.

	**Proportion of aggregated woodlice (%)**	**Proportion of woodlice under shelters (%)**	**Proportion of woodlice outside shelters (%)**	**N=**
40 woodlice	88.2 (± 7.1)	87.1	12.9	29
60 woodlice	87.4 (± 7.5)	87.4	12.6	20
80 woodlice	88.4 (± 7.0)	88.4	11.6	20
100 woodlice	89.1 (± 5.6)	89.1	10.9	18

**Figure 2.**
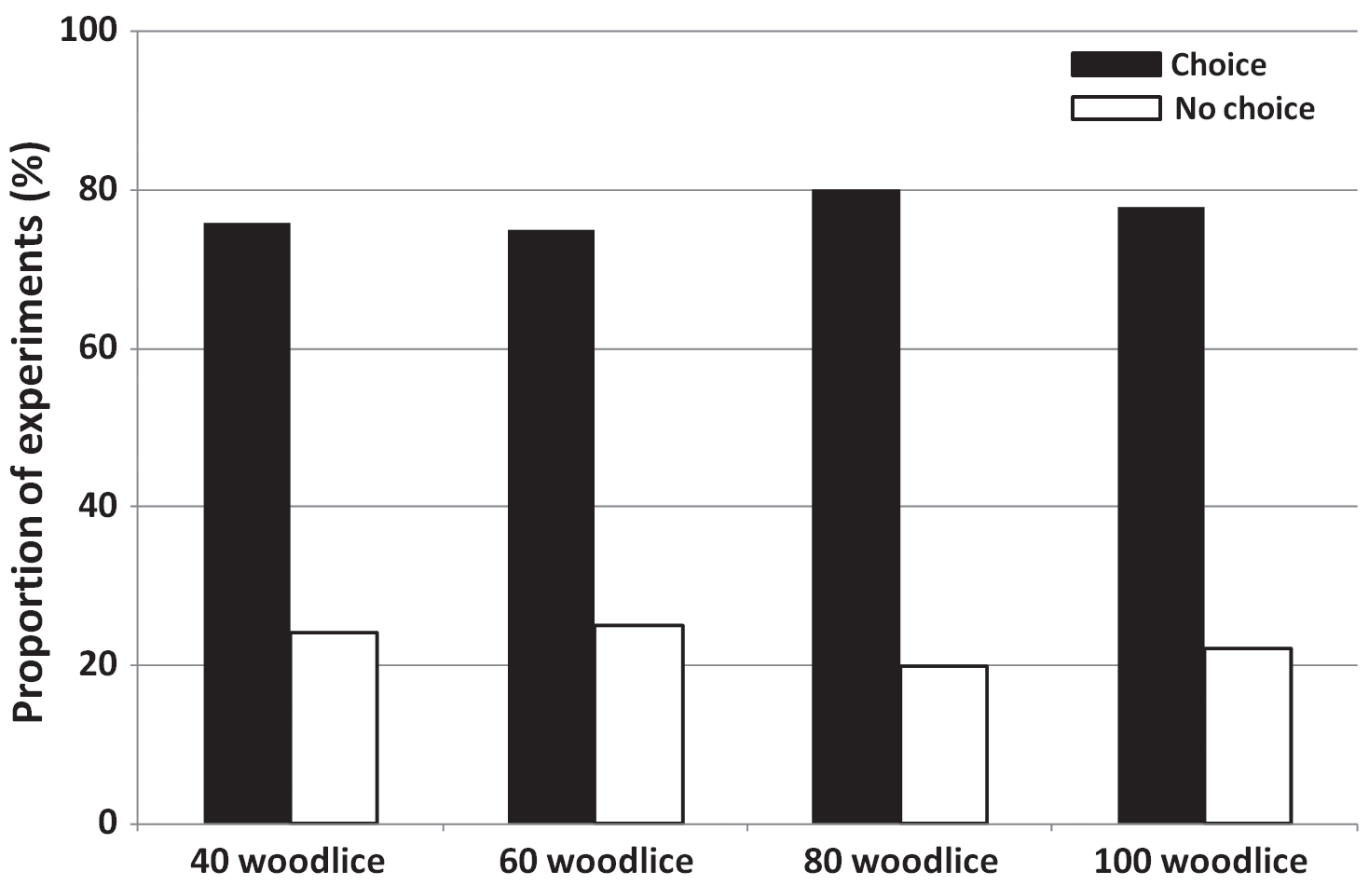
**Choice of one shelter.** Proportion of choice of a shelter at the end of the experiments as a function of woodlice density.

Experiments showed that groups of woodlice generally selected one of both shelters (Fig. 2). Indeed whatever the density condition, more than 77% of all replicates (regardless of isopod density) showed a clear selection of only one shelter (Fig. 2. c² test, c²=0.17, p=0.98 – no difference between density conditions).

In order to understand the aggregation dynamics, separate analyses of replicates with a clear choice of one single shelter (77%, N=87) and replicates where isopods reparted almost equally among the two shelters, i.e. no selection of one shelter (23%) were necessary. However, the number of replicates without choice was low and were evenly distributed among the four densities tested (Fig. 2). Hence, in the remaining part of this paper only replicates with choice will be described and discussed.

**Figure 3.**
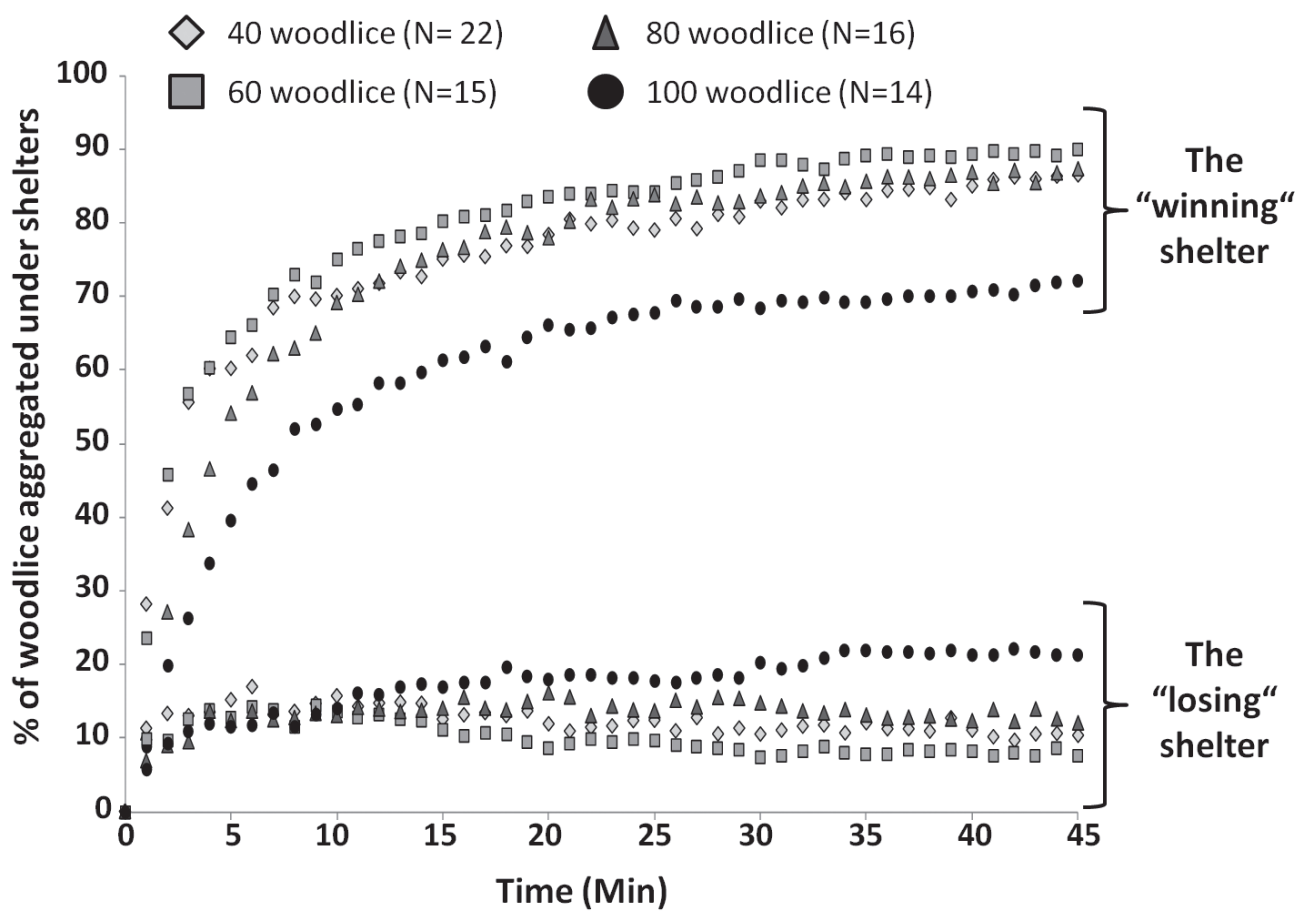
**Dynamics of aggregation under shelters.** Average proportion of woodlice aggregated under the “winning” and the “losing” shelter for experiments showing a clear choice of one of both shelters (Binomial test, difference from an equal distribution of woodlice between shelters).

**Figure 4.**
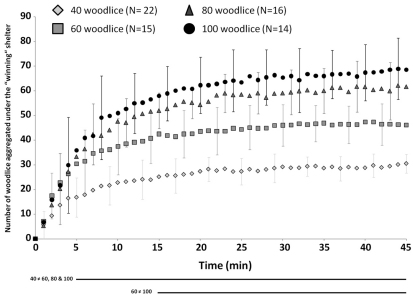
**Dynamics of aggregation under the** “**winning**” **shelter**. Evolution of the average number of woodlice under the winning shelter as a function of time for the four densities tested and for experiments showing a clear choice of one of both shelters. Standard deviations are presented for each 4 minutes. Horizontal lines below the graph indicated the statistical differences between densities; these differences were pointed out by a Kruskal-Wallis followed by a Dunn’s tests for each minute of the experiments.

Woodlice showed a strong thigmotactic behaviour; just after their release, woodlice walked in the arena, generally near the edge and they quickly entered under both shelters (Figure 3). The number of woodlice increased simultaneously under both shelters but, most woodlice quickly concentrated under one shelter (Figure 3). In less than 3 minutes on average, one aggregate was larger under one of the shelters and it remained larger during the experiments. This result was observed at any density condition tested (time of selection was 2.18±1.5, 2.06±2.28, 1.69±1 and 2.14±1.6 minutes for treatments with 40, 60, 80 and 100 woodlice set-ups, respectively; Kruskal-Wallis’ test p=0.42). Whatever the density, the proportion of woodlice under the “winning” shelter quickly increased to more than 50% of woodlice in less than 10 minutes for each treatment (Fig. 3). After 10 minutes, the proportion of aggregated woodlice under the “winning” shelter slightly increased to stabilize at nearly 80% of woodlice for 40, 60 and 80 woodlice experiments and around 70% with 100 woodlice set-up at the end of the experiments (Fig. 3). However, there were significantly more woodlice under the “winning” shelter when there are more woodlice in the set-up except when the number of woodlice is higher than 80 (Fig. 4 – Kruskal-Wallis test). The proportion of aggregated woodlice under the “losing” shelter was around 7–10% of woodlice in 40, 60 and 80 woodlice set-ups (Fig. 3 – average of 10.7±10.7, 7.1±10.8 and 9.1±10.5% for 40, 60 and 80 woodlice set-ups, respectively). This proportion reached 20.4±12.7% in set-ups with 100 woodlice (Fig. 3). The individuals which were not found under the shelters were observed walking in the arena. Whatever the experiments, these walking woodlice generally consisted in 10–15% of population introduced.

## Discussion

The densities used in this study do not impact the aggregation process. Indeed, no differences were observed between density conditions in the dynamics of aggregation, the collective choices and the rates of selection of only one shelter. Aggregation in woodlice is very frequent (Allee 1931; Friedlander 1965). Only one of all 88 replicates did not show any aggregation during 45 minutes. Woodlice aggregation always occurred under shelters, i.e. under reduced light conditions. Small aggregations observed outside shelters were not stable. Hence, individual preferences ruled the location of aggregates and our results confirmed that populations of woodlice were able to select one shelter when two identical shelters were available (Devigne et al. 2011). This study shows, for the first time, that this collective choice is not impacted by woodlice density. With a very high proportion of aggregated woodlice, only 10–15% of individuals were observed walking on the arena at the end of experiments, regardless of the density treatment. Although most of the characteristics of the aggregation did not vary with the density, some particularities deserve a discussion which will point out the complexity of mechanisms which come into play during this phenomenon which have been often tackled but are still not well understood. This discussion will also give rise to new issues for future investigations.

In more than 77% of experiments, a choice of one shelter was made by groups of woodlice. In such experimental conditions, these selections can only be explained by the social interactions between congeners (Camazine et al. 2001, Jeanson and Deneubourg 2007, 2009). However, individual preferences are important since even with higher densities, woodlice never aggregated outside the shelters. These results moderate our first observations which showed that social interactions could outweigh individual preferences in the collective decision making by leading the groups toward suboptimal choices (Devigne et al. 2011). In this respect, observation of systematic aggregation under shelters could be explained by the high density in our experiments. Indeed, the increase of density can enhance the efficiency of collective choice and hence to decrease the frequency of suboptimal choices (Canonge et al. 2011, Sempo et al. 2009). Moreover the expected increase of the selection rate of only one shelter due to higher density was not observed: the same proportion of experiments showed a selection of one shelter whatever the tested densities. At the higher density, the main aggregation reached a plateau (around 70 woodlice in our conditions) and most of the other woodlice were found under the second shelter. Hence the lack of increase of selection rate with density could result from a saturation of the selected shelters (see below).

Concurrently to the absence of an enhanced selection rate, our results did not show the expected acceleration of aggregation dynamics, in conjunction with higher density. Indeed, this phenomenon being driven, in part, by social interactions between congeners, aggregation in a preferred shelter should happen faster at higher densities. In our experiences, whatever the number of woodlice, the aggregation was very quick (in less than 10 minutes most of woodlice are aggregated) and did not differ between densities. Possibly, the aggregation process was already very quick even at our lowest density used (1325 individuals/m² corresponding to 40 individuals) so that the phenomenon could not happen any faster. The density used in these experiments corresponded to the high values observed in nature (Gongalsky et al. 2005, Paoletti and Hassall, 1999) but lower densities are often observed in the field. More investigations with lower densities and also at other spatial scales –density-dependence relations can vary according the spatial scale (Courchamp et al. 2008)– should allow us to identify the relative part of individual preferences and social interaction in the aggregation dynamics and better understand the diversity of aggregation patterns.

The number of woodlice aggregated under the “winning” shelter increased with the number of woodlice within the setup. However, from a number of 80 woodlice in the set-up, the number of woodlice under the “winning” shelter reaches a plateau around 70 woodlice (no difference was found between 80 and 100 woodlice set-ups). This result firstly suggests a saturation of shelters at 70 woodlice. This may result from the shelter carrying capacity. Nevertheless, a stable aggregation under a shelter whatever the density, often extended beyond the edge of that shelter. As a consequence, some woodlice belonging to the aggregation were not in the darker area. Keeping in mind that at the 100 woodlice condition, a second stable aggregate grows under the losing shelter, two “functional” hypotheses, deserving new investigations, can explain this maximal number of woodlice in an aggregate. Firstly, it is possible that competition in the aggregate increases with the number of woodlice and beyond 70 woodlice, it could be better for a woodlouse to join a smaller aggregate (Brereton 1956, Lefebvre 2002, Paris 1963, Thiel 2011). Secondly, the benefits of aggregation concerning the reduction of water loss could decrease at large cluster size (Allee 1926; Cloudsley-Thompson and Constantinou 1987, Edney 1951, Gunn 1937, Warburg 1964). Indeed, from a size of around 60–70 aggregated individuals, the woodlouse did not reduce their water losses (Kuenen and Noteboom 1963, Broly et al. In prep). Therefore it could try to join another smaller aggregate but with the opportunity to be under a shelter. Besides, aggregates with more than 70 woodlice were sometimes observed. However, the activity of woodlice was higher and these aggregates are transitory and hence unstable.

These results were in accordance with the existence of aggregation pheromone coming from faeces suggested by the past (Kuenen and Noteboom 1963, Takeda 1984). However the speed of the aggregation (in 10min) questions about the implication of such potential pheromone coming from faeces. Indeed during the experiments, woodlice produce a small amount of faeces. Other pheromones released by individuals could potentially be involved in the aggregation process. However specific experiments dealing with the implication of pheromone in aggregation process currently occur to decipher the part and the role of such signal during the aggregation in *Porcellio scaber*.

In the field, in woodlice and most of the organisms, the local population densities depend on characteristics of their environment (litter, Zimmer and Topp 1997; temperature or humidity, Zimmer and Brauckmann 1997). Moreover, in the species social interactions can impact the spatial distribution by promoting aggregated patterns. Better knowledge about aggregation processes and measures of density at small scales would allow us to understand their spatial distribution in nature (Detsis 2009, Grear and Schmitz 2005, Kao 1984, Tremblay and Gries 2006). Our results showed that location of aggregates is strongly governed by individual preferences and that the dynamics of aggregation and collective choice are controlled by social interaction between congeners. Nevertheless, densities did not impact the aggregation patterns. That could seem surprising because when the number of woodlice increases, potential interactions increase too and dynamics should be modified. Maybe the densities chosen in our experiments were too high to observe any change. However, our results showed that maximum number of woodlice in a cluster is reached in high density conditions. If our results showed a maximum number around 70 woodlice in a cluster, besides shelter size this value certainly depends on environmental conditions (e.g. humidity) or physiological state of woodlice inside aggregates.

Moreover, a complete understanding of the woodlice aggregation and its characteristics needs a theoretical approach of the costs and benefits of the aggregation in order to evaluate the differences for woodlice between optimal and stable sizes of clusters (Krause and Ruxton 2002, Sibly 1983, Thiel 2011).

Social interactions in woodlice and different environmental parameters (such as maximum carrying capacity of shelters or maximum size of aggregates) are important to understand the distribution of woodlice in the environment. In natural conditions, a local peak of population (in case of binary choice, the population is higher on one side) may result from the coupling between the response to the environmental heterogeneities and the social interaction. Moreover, even if more investigations are necessary to decipher the mechanisms explaining, the velocity of gathering in aggregates, the maximum size of clusters and the social signals used we suggest that similar observations could be made now in field.

Since woodlice are often used as bioindicators for pollution, the explanation of the collective decision making and patterns of aggregation of woodlice population could inform us about quality of environment (Godet et al. 2011, Loureiro et al. 2006, Zidar et al. 2004, 2005) and could improve experimental tests used to assess soil contamination (Kaschl et al. 2002, Loureiro et al. 2005, Zidar et al. 2002). The social interaction, amplifying the individual response, could explain the disagreement between the response of isolated individuals and the response of a group as suggested by Loureiro et al. (2005). If group size effect on the survival rate are well-known (Allee effect, Brockett and Hassall 2005), future studies on choice, preference and avoidance could take account these social effects and the size of the tested population (at least isolated individuals vs groups) that could affect the experimental results and the conclusions.
